# Region-Based CNN for Anomaly Detection in PV Power Plants Using Aerial Imagery

**DOI:** 10.3390/s22031244

**Published:** 2022-02-07

**Authors:** Michiel Vlaminck, Rugen Heidbuchel, Wilfried Philips, Hiep Luong

**Affiliations:** 1 IPI-URC-imec, Ghent University, Sint-Pietersnieuwstraat 41, 9000 Ghent, Belgium; wilfried.philips@ugent.be (W.P.); hiep.luong@ugent.be (H.L.); 2 Sitemark, Gaston Geenslaan 11, 3001 Leuven, Belgium; rugen.heidbuchel@sitemark.com

**Keywords:** anomaly detection, UAV, inspection, PV power plants, deep learning, thermal imaging

## Abstract

Today, solar energy is taking an increasing share of the total energy mix. Unfortunately, many operational photovoltaic plants suffer from a plenitude of defects resulting in non-negligible power loss. The latter highly impacts the overall performance of the PV site; therefore, operators need to regularly inspect their solar parks for anomalies in order to prevent severe performance drops. As this operation is naturally labor-intensive and costly, we present in this paper a novel system for improved PV diagnostics using drone-based imagery. Our solution consists of three main steps. The first step locates the solar panels within the image. The second step detects the anomalies within the solar panels. The final step identifies the root cause of the anomaly. In this paper, we mainly focus on the second step comprising the detection of anomalies within solar panels, which is done using a region-based convolutional neural network (CNN). Experiments on six different PV sites with different specifications and a variety of defects demonstrate that our anomaly detector achieves a true positive rate or recall of more than 90% for a false positive rate of around 2% to 3% tested on a dataset containing nearly 9000 solar panels. Compared to the best state-of-the-art methods, the experiments revealed that we achieve a slightly higher true positive rate for a substantially lower false positive rate, while tested on a more realistic dataset.

## 1. Introduction

Photovoltaic (PV) power plants are playing a fundamental role in supporting the transition to a green economy and achieving energy and climate objectives. However, degradation of solar panels over time can have a high impact on the performance of PV sites. Therefore, PV plants need to be regularly inspected for defects. Unfortunately, the latter is a very time-consuming and sometimes dangerous task and often goes hand in hand with a cessation of the energy production. All this means that frequent inspections are not very cost effective and site operators need more advanced tools for fault localization, power loss quantification and improved diagnostics of PV plants. A more efficient detection, diagnosis and localization of the fault will not only lead to a more cost-effective inspection but will also reduce the time during which the fault can impact production.

The work presented in this paper aims at developing image processing techniques that exactly serve this purpose. More specifically, we developed algorithms to automatically identify anomalies and determine their root causes using drone-based imagery. To achieve this goal, we combined data from two different modalities, namely an RGB camera and a thermal camera. The combination of both sensors is crucial. On the one hand, many defects are visible in thermal images as they provoke an increase in temperature in the respective solar panel, such as hotspots. On the other hand, the thermal image itself does not provide information on the root cause of the anomaly. This is where the RGB image comes into play as it can help us determine the root cause. For example, some hotspots are provoked by external factors such as overgrown vegetation, droppings from birds or the accumulation of dirt and dust better known as soiling. In some cases, no peculiarity can be observed in the RGB image, which probably means that the root cause is not external. This may indicate a more severe underlying defect such as an issue with the junction box or diode. This information is critical for the operator as it allows him to make the right decision on whether to send a mechanic in the field to fix the defect or to initiate a cleaning procedure.

Today, no suitable PV diagnostics tool is available for the PV industry that provides accurate and reliable information on defects in an automated manner. On the one hand, some tools exist to detect and quantify under-performance issues based on the observation of current, voltage or power [1,2]. However, while they are well-suited for detecting degradation within a PV module or array, they are less suited for detecting faults or defects on the solar panel level. As a result, it remains time consuming to locate the anomalies and identify their root causes. The popular technique of aerial electroluminescence (EL) [3,4] has the same drawback.

For that reason, it is beneficial to use infrared thermography to accurately identify and locate faults and defects in PV plants. Unfortunately, many techniques for PV inspection that use thermographic images acquired by drones are based on the analysis of the temperature distribution [5,6,7]. The main problem with these is that they suffer from thermal drift or varying ambient temperatures making them less reliable. Finally, with the increasing interest of artificial intelligence in all kinds of domains, some AI-based solutions for PV site monitoring were recently presented. They generally use deep convolutional neural networks and show promising results. However, the main drawback of the solutions presented so far in literature is that they lack ‘generalizability’ and as a result they perform poorly on ‘previously unseen’ PV plants, making it necessary to retune the (hyper)-parameters for each newly scanned PV site. The latter makes the entire process again time consuming. In this work, we aim at automating PV inspection by increasing accuracy and reliability, hence reducing the total manual inspection time.

The contributions of this work are two-fold. First, we developed a novel anomaly detector for thermal images achieving unprecedented accuracy. The main novelty and success factor lies in our three-stage approach: (1) generating an orthophoto, (2) pre-locating the solar panels and, finally, (3) detecting the actual anomalies. The first step allows us to apply appropriate scaling of the thermal data (dealing with thermal drift), whereas the second step reduces the region of interest for potential anomalies and, thus, mitigates the risk for false positives appearing in regions of non-interest. Note that the solar panel detection or localization is not strictly necessary but contributes to a more accurate detection of anomalies. Second, we propose an appropriate normalization of the thermal images in order to make the appearance of solar panels and anomalies more uniform across the orthophoto from a single PV site as well as across orthophotos from multiple PV sites.

## 2. Related Work

Several existing systems for PV inspection are monitoring current, voltage or power to detect anomalies [1]. Akiyama et al. [2] developed a power line communication method that utilizes direct current (DC) lines, which has made it possible to monitor every solar panel in a PV plant. Another popular technique is aerial electroluminescence (EL) imaging, which is currently the most cost-efficient method on the market for the early detection of solar systems affected by potential-induced degradation (PID). One company that is providing EL imaging as a service is *Quantified Energy Labs* (https://qe-labs.com/, accessed on 12 September 2021). However, EL imaging, compared to infrared (IR) imaging, has its limitations. Infrared imaging can provide the exact physical location of the defect not only on the level of a solar module or panel but also within a solar cell. It is, thus, more suitable to perform a quantitative and qualitative diagnosis of the PV plant. For that reason, the company ‘*Aerial PV Inspection*’, in short AEPVI (http://www.aepvi.com, accessed on 12 September 2021 ), developed a system that uses a combination of infrared (IR) and EL imaging and machine learning to identify faults. The AEPVI company was not the first to explore the use of thermal imaging for photovoltaic inspection. More research on this topic was published lately, which can roughly be divided in two separate categories: (1) methods based on classical image analysis techniques and (2) methods based on deep learning.

In the first category, Tsanakas et al. [8] proposed to apply classical image segmentation techniques on thermal images, such as the Canny Edge detector, and image histogram analysis to identify faults and quantify them in terms of relative area size and number of impacted cells. However, their solution was only tested on two PV sites for which they claim to diagnose 13 out of 14 and 27 out of 29 defective cells, which is statistically insignificant. Kim et al. [9] designed a similar system for detecting malfunctioning PV cells using drone-based thermal imaging. They use statistical analysis on the thermal intensity characteristics (e.g., surface temperature) of each PV module. More specifically, they determined the mean intensity and standard deviation of each panel as parameters for fault diagnosis. The authors claim a detection accuracy for defective panels of 97%, but their algorithm was only tested on three sample images.

Rogotis et al. [5] developed yet another PV defect detection algorithm based on classical techniques. They adopt a two-step approach in which they first localize the PV modules using image segmentation based on Otsu thresholding. Subsequently, they utilize a global threshold computed by a combination of two thresholding techniques ‘*Mean Relative Thresholding (MRT)*’ and ‘*Mean Frequency Thresholding (MFT)*’. Finally, they apply morphological dilation and test the candidate defected areas using three criteria based on size, shape and intensity distribution. Gao et al. [6] also presented an algorithm that segments the solar panels from the background prior to the detection of anomalies. They claim that the segmentation runs in real-time and that only the height of the array is required as prior information to aid in the segmentation process. Hot panels are subsequently detected using DBSCAN clustering. Finally, Buerhop et al. [7] present their aIR-PV-check solution, a system using unmanned aerial vehicles (UAV) equiped with an infrared (IR)-camera for the quality inspection of PV-plants under real operating conditions. Similarly to many of previously described solutions, defective modules are detected by their temperature distribution.

Even though some of the aforementioned methods based on the histogram or distribution of temperature achieve a good detection performance on the evaluated sites, these solutions lack generalizability. For example, they are largely affected by different ambient temperatures and they struggle when other areas within the PV module are significantly overheated. Moreover, sometimes multiple hotspots can be present within one solar panel, or different hotspots can have different delta temperatures. As such, the histograms might have more than one peak, making it more difficult to define an adequate threshold. Due to these reasons, most recent research studies focus on the application of deep neural networks to detect and locate anomalies and defects within photovoltaic power plants.

In the second category of deep learning based methods, Pierdicca et al. [10] trained a VGG-16 network for the automatic detection of damaged photovoltaic cells in thermal images. They apply the network on images that are resized to 224 × 224 pixels without pre-determining a region of interest. The network, thus, acts as a classification network that assigns a binary label depending on whether or not a defect is present in the image. The presented approach is, therefore, very limited. First, it does not locate the precise defect within the image; thus, the operator does not know where the defect occurs. Second, the model is highly influenced by varying ambient temperatures and various backgrounds in the images.

Pierdicca et al. [11] improved their PV fault detection algorithm. This time, they perform object detection (and thus localization) on top of classification. To that end, they have trained a mask region-based convolutional neural network (CNN) to detect anomalous solar panels. In this work, we will use a similar approach based on a region-based CNN, but in contrast to [11] where one CNN was trained to detect anomalous solar panels, we adopt a two-stage approach in which we first train a CNN to detect all solar panels in the orthophoto of the PV site and another CNN to subsequently detect the anomalies. In other words, we detect anomalies within solar panels rather than we detect anomalous solar panels. We believe that our approach is more effective in preventing the neural network to detect solar panels without a defect and, hence, in reducing the amount of false positives. Note that our approach still allows the detection of anomalies without priorly detecting solar panels, albeit with reduced accuracy.

Oliveira et al. [12] combined a traditional image segmentation technique with deep learning based classification. First, they segment the defected solar panels by using Laplacian-based edge detection. Subsequently, they train a VGG-16 network to classify the defect into three categories: disconnected substrings, hot spots and disconnected strings. The same authors later on extended their fault detection system by incorporating an RGB camera to further differentiate between physical defects and hot spots caused by soiling and vegetation. In our system we also integrated an RGB camera next to a thermal camera to allow for more precise root cause analysis. We will briefly describe the workflow of this analysis later on in this paper, but we plan to elaborate on this in a future publication. Finally, Alves et al. [13] developed a system that classifies the anomalies between up to eleven different classes solely based on thermographic images.

One of the drawbacks of existing solutions is that they remain vulnerable when thermal drift is occurring or when the ambient temperature is varied. This means that the proposed solutions from the literature lack generalizability. Our solution can deal with the aforementioned challenges thanks to several improvements that we propose. First, we apply appropriate scaling of the thermal image. Second, we train our AI model using several data augmentations including random brightness, lighting and saturation. Third, we adopt a three-step approach by first detecting solar panels in a thermal-normalized orthophoto—which is an easier task—and subsequently identifying anomalies within the solar panels. As a result, our combined AI model generalizes very well and can operate on different PV sites in a trustworthy manner.

## 3. UAV-Based PV Anomaly Recognition System

The entire work-flow consists of several algorithms and can be summarized into five main steps:

1. Structure from motion and registration of RGB and thermal imagery;
2. Detection of solar panels;
3. Detection of anomalies in thermal images;
4. Determination of the root cause of the anomaly using RGB and thermal images;
5. Estimation of the shading profile based on the 3D model.

The algorithms related to steps 1 and 2 were previously described in [14,15]. In this paper, we will focus on the detection of anomalies using thermal imagery (step 3). The algorithms related to the root cause analysis (step 4 and 5) will only be briefly explained. The techniques and experiments will be discussed in detail in Section 5 and Section 6. For the sake of completeness, we will summarize all steps in this section.

### 3.1. Structure from Motion and Registration of RGB and Thermal Imagery

The first step of the workflow deals with the generation of an accurate georeferenced orthophoto and 3D model of the PV site by means of structure from motion or photogrammetry. This step is necessary to be able to relate each damaged solar panel from the image to the corresponding solar panel in the physical world and in the digital twin of the solar plant. As the environment consists of highly repetitive patterns, due to the abundance of solar panels, matching key points between RGB images is far from trivial as one has to deal with ‘multi-correspondences’ or in some cases even shifted sets of correspondences. To cope with these challenges, we have developed an improved matching algorithm [14]. The matching is based on pixel-distance and pixel-rotation histograms derived from GPS data to estimate an initial guess on the translation and orientation (only two orientations are considered, 0° and 180° with respect to the initial orientation) made by the drone. All keypoint matches that agree with the initial translation and orientation are selected and used to estimate the camera pose. The other matches are discarded, resulting in more accurate pose estimates. We refer the reader to [14] for more details.

The orthophotos are generated for both the thermal and RGB images and are subsequently aligned. In performing this, we obtain every solar cell and panel visual as well as thermal information, which will be used in the subsequent steps of anomaly detection and root cause analysis. In addition, the 3D model obtained by structure from motion will be used to derive the shading profile of the PV site. The shading information will on its turn allow the prediction of the expected power output at the solar panel level and to compare it with the true power output derived from monitoring data.

### 3.2. Detection of Solar Panels in Thermal Images

One of the prerequisites for an accurate and efficient detection of anomalies is the precise localization of the solar panels. Once we know their precise location, the anomalies can be more accurately detected as its search is restricted to the specific region of the solar panel in the thermal image. This two-step approach is not only faster, but it also mitigates the risk of detecting false positive anomalies. For the detection of the solar panels, we rely on RGB imaging because of the much higher spatial resolution and color information, but also on the thermal data. The borders of the solar panels are generally colder than the panels themselves and appear darker in thermal images, which make them well suited for the task.

**Figure 1:**
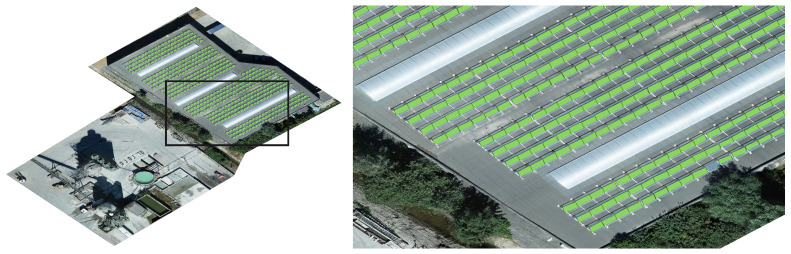
Example of solar panel detection on a Belgian, rooftop-based PV site (**left**) and a zoomed in version (**right**). The solar panel detection is used to reduce the search window for the anomaly detection algorithm.

The procedure for the detection of solar panels is extensively described in [15]. In summary, two different methods were developed. One method is based on classical image processing techniques (including edge detection) and is used to generate training data in an efficient manner. The second method is based on the training of a region-based convolutional neural network (Mask-RCNN). An example of the solar panel detection on a Belgian rooftop-based PV site is depicted in Figure 1.

### 3.3. Detection of Anomalies in Thermal Images

**Figure 2:**
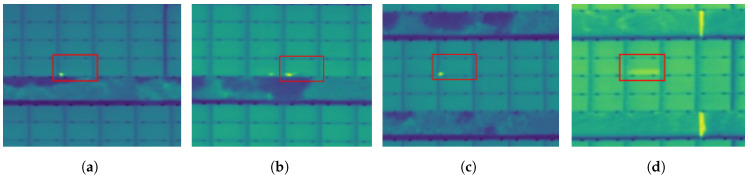
Three hotspots (**a**–**c**) and a bypass substring (**d**) in a 16-bit thermal image, here depicted using a two-channel image (using the ‘blue’ and ‘green’ channel).

In thermal images, two different anomalies can be distinguished: (1) hotspots and (2) bypass substrings, both provoking temperature increase. Hotspots appear as high-intensity blobs in a thermal image, whereas bypass substrings appear as elongated blobs that cover one-third of the solar panel or two-thirds in case of a double bypass substring. In Figure 2, a few examples of a hotspot and an example of a bypass substring in a thermal image are depicted. In this work, we mainly focus on the detection of hotspots as they represent the vast majority of the anomalies present in operational PV plants. To achieve our objective, we use an adapted version of the Faster R-CNN model [16]. The latter is an object detector and hence fulfills the combined task of both image classification and object localization. As such, it takes an image as input and produces one or more bounding boxes with the class label (in our case we use a binary label) attached to each bounding box. Strictly speaking, the prior detection or segmentation of solar panels is not necessary, but nonetheless it will reduce the region of interest and as such mitigate the risk of false positives. On the other hand, the object detector will still properly work when the solar panels are inaccurately segmented.

### 3.4. Determination of the Root Cause of the Anomaly Using RGB and Thermal Images

**Figure 3:**
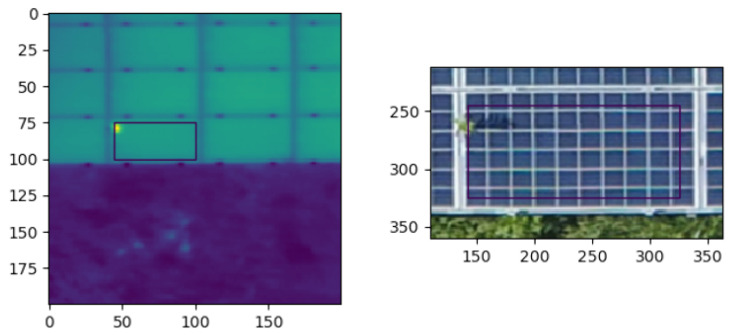
Hotspot in a thermal image (**left**) caused by overgrown vegetation visible in the RGB image (**right**). While thermal images can be used to easily detect hotspots, they do not provide information on the root cause of the anomaly. The RGB image can help to determine the latter anomaly: in case a peculiarity is visible, the hotspot may be caused by an external factor, whereas in the other case the cause is probably physical, indicating a more severe underlying defect.

Once a hotspot is detected in the thermal image, we inspect the region in the RGB image that corresponds to the affected solar panel. In case the hotspot is induced by an external factor, the RGB image will most likely show a peculiarity, as seen in Figure 3. To that end, we have developed a novel method based on an autoencoder to detect anomalies in RGB images. The autoencoder is learning to replicate the most salient features of a healthy solar panel in the training data. In other words, it learns to precisely reproduce the most frequently observed characteristics of a solar panel. At inference time, the autoencoder will be able to accurately reconstruct “normal” data, while failing to do so with unfamiliar anomalous data. The reconstruction error can then be used as a score to detect anomalies.

Once an anomaly is detected in the RGB image, we further differentiate between vegetation-induced hotspots, hotspots resulting from (bird) droppings and hotspots resulting from shading. For the latter, we will rely on the 3D model generated by the structure from motion system, as explained in Section 3.5. We are aware that there are other causes of hotspots, such as soiling, corrosion or backsheet cracking. If they result in a peculiarity in the RGB images, they will be detected by the autoencoder but we will consider them as a residual class.

In case the RGB image does not show any peculiarity, while a hotspot is still detected in the thermal image, this may indicate that the cause is a physical failure, for example an issue with a junction box or diode. The further classification into a junction box or diode issue or anything else falls beyond the scope of this work. In that case, the precise root cause should be determined by inspecting monitoring data, e.g., DC or inverter data, or by sending an expert in the field.

### 3.5. Determination of the Shading Profile

**Figure 4:**
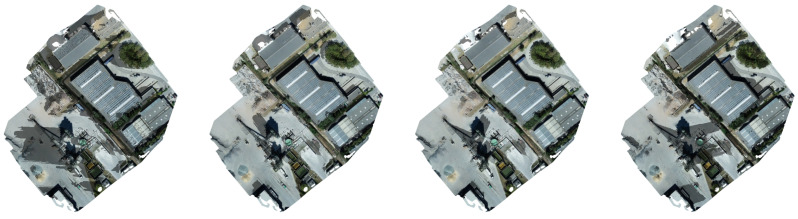
Shadow maps generated for a Belgian PV site for 7 August at times 7:00 AM, 10:00 AM, 1:00 PM and 4:00 PM. By modelling the shading profile during the entire year, we can compare the expected power output with the true output of the respective solar array or string. A discrepancy between both values may indicate that a defect is occurring or that the string or panel array is suffering from degradation.

The final step in the pipeline is to determine the shading profile of the PV plant. By modelling which parts of the solar plant are shaded or lit during the entire year, we can compare the expected power output with the true output of the respective solar array or string. In case both values do not match, this may indicate that a defect is occurring or that the string or panel array is suffering from degradation. The shadow maps will also be used to determine whether the appearance of hotspots in the thermal image could be induced by shading. Figure 4 depicts four shadow maps generated for a small Belgian PV plant during 7 August (the day of the flight) at times 7:00 AM, 10:00 AM, 1:00 PM and 4:00 PM.

## 4. PV Dataset Generation

In order to train our CNN model to detect hotspots in thermal images, we use data from six different PV power plants located in Italy, Spain and Japan. The datasets are highly diverse in many respects. First, they cover both rooftop-based PV sites and ground-based PV sites. Second, they contain several types of solar panels with various appearance and placed under different tilt angles. Third, the datasets were captured under various flight conditions. Some flights were carried out using flight lines that were perpendicular or parallel to the solar bank orientation, to which we will refer as a *vertical* or *horizontal* orientation respectively. Other flights were carried out using flight lines that had another orientation than the orientation of the solar banks, referred to as *oblique* orientation. Fourth, flights were also captured under varying weather conditions. In that respect, sun reflections are present on some parts of the PV site, whereas other areas are covered by shadows from clouds or nearby buildings.

**Figure 5:**
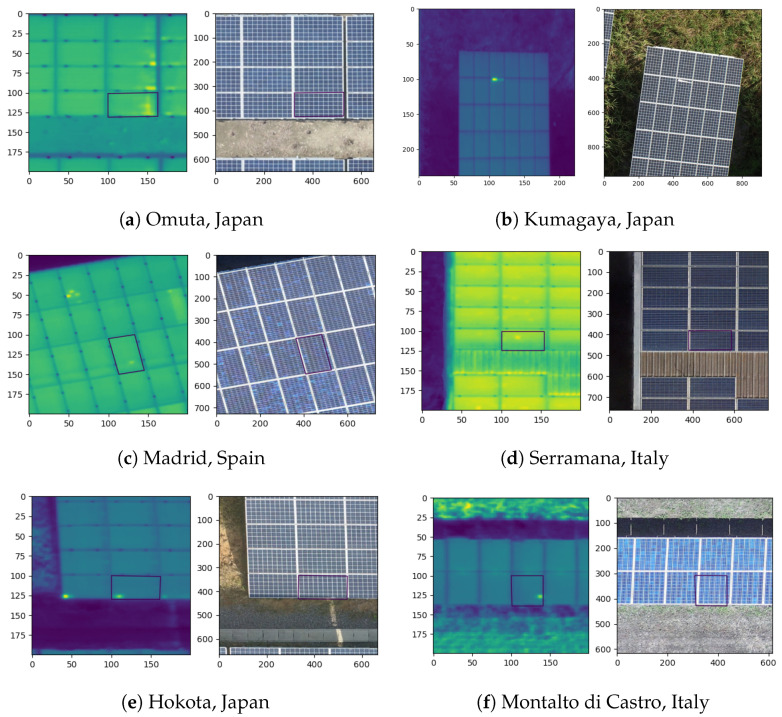
Images from the different datasets, captured at various PV plants in Italy, Spain and Japan. The aggregated dataset is highly diverse, containing ground-based and rooftop-based PV sites. Flights were conducted using flight lines that are parallel, perpendicular or oblique to the orientation of the photovoltaic arrays.

The aggregated dataset is, thus, very complex and it is not trivial to train a CNN that generalizes well, achieves high accuracy and at the same time does not over-fit the dataset. Moreover, many sites also suffer from multiple hotspots appearing on the same solar panel. This motivates the choice to train a neural network instead of relying on histogram or thresholding based methods. In Table 1, an overview of the specifications and number of hotspots for the different PV sites is listed. In Figure 5, a few images of the same PV sites are depicted.

### 4.1. Positive Samples

The ground truth was manually annotated by the operator on the solar panel level. As the goal is to detect anomalies within solar panels, we additionally perform a precise segmentation of the hotspot within the solar panel. In order to achieve this, we use the SLIC (*Simple Linear Iterative Clustering*) algorithm to generate a segmentation mask for the hotspots. The SLIC algorithm clusters pixels based on both their intensity similarity and their proximity in the image plane. The clusters with the highest average intensity values that are also above a certain threshold are considered as hotspots. The segmentation obtained by SLIC clustering was still assessed by a person and only clear examples are accepted in the dataset. In addition, we only keep the hotspots that are sufficiently salient be selecting only those with a delta temperature of at least 5°.

### 4.2. Negative Samples

In order to avoid class imbalance and over-fitting of the CNN model, we guaranteed that a similar amount of negative samples was incorporated into the training dataset. To generate those negative samples, we use our solar panel detection algorithm, which was described in [15].

**Figure 6:**
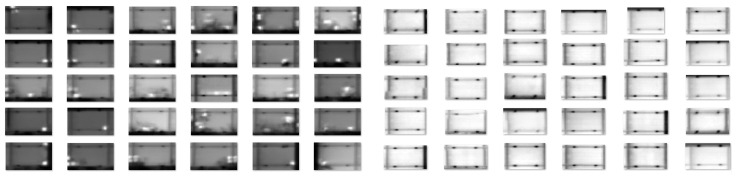
Positive samples of hotspots (**left**) and negative samples (**right**) from our aggregated PV dataset, here depicted as grayscale images.

The final images used for training—both positive and negative—were additionally padded with a few pixels from the surroundings of the solar panel. In Figure 6, a few examples of both positive and negative samples from the training dataset are depicted.

## 5. Proposed CNN-Based Solution for Anomaly Detection in Thermal Images

**Figure 7:**
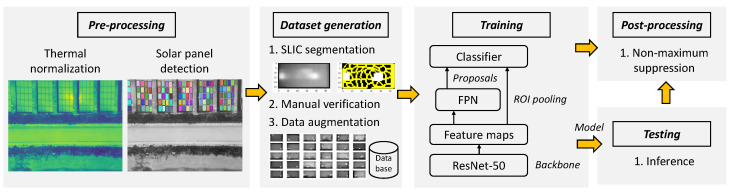
The workflow of our anomaly detection algorithm based on thermal images. In the pre-processing phase, the thermal images are normalized and the solar panels are detected. The latter are fed to SLIC segmentation to generate positive samples of hotspots. These are manually checked and the dataset is augmented using several operations (rotations, crops, contrast, etc.). Training is based on a region-based CNN, using a Feature Pyramid Network (FPN) as region proposal network and a ResNet-50 as backbone. Finally, post-processing comprises non-maximum suppression to filter out ‘duplicate’ (i.e., overlapping) detections.

In this work, we aim at precisely identifying the anomaly within a solar panel and within the entire PV site. The output of our algorithm is a bounding box comprising the anomaly located in a georeferenced orthophoto. The workflow of our solution is depicted in Figure 7.

### 5.1. Pre-Processing

As an orthophoto typically contains a large number of pixels, depending on the actual size of the site, we tile it in smaller images of 500 by 500 pixels. Then, we segment the different panels using a trained Mask R-CNN network [17], cfr. our work presented in [15]. The panel segmentations are eventually used as a prior for the location of potential anomalies. In other words, we use segmentation masks to reduce the search window for the anomaly detection algorithm. The latter not only limits computation time, but it also guarantees a more robust detection of defective solar panels as similar ‘thermal blobs’ in other regions will not result in false positives. As a result, the threshold on the confidence score can be more finely adjusted, resulting in both higher recall and precision values.

As the panel segmentations are not perfect, we use a padding of eight pixels to guarantee that the entire solar panel, including the borders, is visible. This is particularly desired when the hotspot is appearing at the edge of the solar panel. In addition, we made sure that a part of the ‘background’ is visible in order to provide the neural network with sufficient context information.

The thermal data from the FLIR camera outputs 16-bit grayscale images. We normalize the data in such a manner that it fits in a single 8-bit channel. One method to perform this is to use min-max normalization to squeeze the data into the range of 0 to 255. However, as we tile the images in 500 by 500 pixels, it might happen that some images only contain solar panels, whereas other images contain solar panels together with a part of the background, e.g., grass or a concrete roof, both having different properties. As a result, min-max normalization will be highly influenced by the minimum and maximum values, and it will be biased based on where the image has been tiled.

For that reason, we scale the data from the entire orthophoto beforehand. However, we also experienced thermal drift during acquisition and, hence, the ortophoto suffers from uneven illumination. Moreover, the presence of clouds or sun reflections causes the orthophoto to be unevenly illuminated. For those reasons, we apply robust scaling on the thermal images by using statistics that are robust to outliers. This means that in order to compute the mean and standard deviation, we first remove the ‘outliers’ from the dataset. We then scale the data according to the quantile range. For example, the interquartile range (IQR) denotes the range between the 1st quartile (25th quantile) and the 3rd quartile (75th quantile). This scaling is, therefore, not influenced by a small number of very large marginal outliers and consequently, the resulting range of the transformed thermal values is larger than for min-max scaling. Therefore, we use the median value of the image *I* and the 1st quartile $Q_{1}$ and 3rd quartile $Q_{3}$ to compute normalization for each pixel *i* according to the following formula:

(1) $$i^{\prime }=\frac{i-Q_{1}\left(I\right)}{Q_{3}\left(I\right)-Q_{1}\left(I\right)},$$

where the final intensity is $i^{*}=\max\left(0,\min\left(255,i^{\prime }\right)\right)$.

### 5.2. Region-Based CNN Model

Within one solar panel, there can be more than one hotspot present and we do not know beforehand how many exactly. For that reason we cannot simply solve the problem by building a standard convolutional neural network (CNN) followed by a fully connected layer as the length of the output layer is variable and not constant. A naive approach would be to take different regions of interest from the image and use a CNN to classify the presence of an anomaly within each region. The problem with this approach is that the anomaly can have different spatial locations and different aspect ratios. One would, thus, have to select a large number of regions, resulting in a tremendous amount of computation time. For that reason, we decided to use a region-proposal CNN (R-CNN) [18] network, which was developed to find multiple occurrences in a faster manner.

The main drawback with the original R-CNN method of [18] is that it performs a forward pass for each object proposal without sharing computation with the detection network. In [19], the authors, therefore, propose Fast R-CNN in which the network first processes the entire image with several convolutional and max pooling layers to produce a convolutional feature map. Then, selective search is applied to determine object proposals, and for every proposal, a fixed-length feature vector from the feature map is extracted using a region of interest (RoI) pooling layer. This feature vector is subsequently fed into a sequence of fully connected layers that finally result in two output layers: one that produces probability estimates over the object classes and another layer that outputs four real-valued numbers denoting the bounding-box positions for one of the classes.

**Figure 8:**
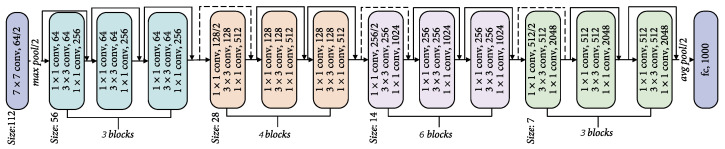
The architecture of the ResNet-50 model [21], used as backbone for our Faster R-CNN based anomaly detection method. Image adapted from [22].

The Fast R-CNN method still has the limitation that it uses selective search to determine the region proposals. In addition to the fact that selective search is slow, it also misses the opportunity to allow the network learn region proposals. For that reason, the authors of [16] proposed their Faster R-CNN method, where region proposals are predicted by using a deep neural network that shares convolutional layers with the object detection network, called the region proposal network (RPN). The marginal cost for computing them is, therefore, small. RPN is designed to efficiently predict region proposals with a wide range of scales and aspect ratios. To that end, a sliding window is used, and at each sliding-window location, multiple *anchors* are considered, an anchor being associated with a scale and aspect ratio. The original Faster R-CNN used anchors of multiple pre-defined scales and aspect ratios in order to cover objects of different shapes. In our solution, we use the Feature Pyramid Network (FPN) as was presented in [20]. We use anchors of three different aspect ratios 1:2, 1:1, 2:1 at each pyramid level. When Faster R-CNN was first published, the authors presented their results using ZF and the VGG-16 as detection networks. In our solution, however, we use the more powerful ResNet with 50 layers (ResNet-50) as the base model. The architecture of this network is depicted in Figure 8.

### 5.3. Loss Function

Regarding the RPNs, we assign a binary class label (being an anomaly or not) to each anchor. We assign a positive label to two kinds of anchors: the anchor with the highest intersection-over-Union with a ground-truth box or an anchor that has an IoU overlap higher than 0.5 with any ground-truth box. The loss function is given by the following equation.

(2) $$L\left(p_{i},t_{i}\right)=\frac{1}{N_{cls}}\sum_{i}L_{cls}\left(p_{i},p_{i}^{*}\right)+\lambda \frac{1}{N_{box}}\sum_{i}p_{i}^{*}L_{box}\left(t_{i},t_{i}^{*}\right).$$

In this equation, *i* is the index of an anchor and $p_{i}$ is the predicted probability of anchor *i* being an anomaly. The groud-truth label $p_{i}^{*}$ is 1 if the anchor is positive, and it is 0 if the anchor is negative. $t_{i}$ is a vector representing the four parametrized coordinates of the predicted bounding box (x,y,w,h), and $t_{i}^{*}$ is the one of the ground-truth box associated with a positive anchor. The classification loss $L_{cls}$ is the log loss over the two classes (anomaly vs. not anomaly). For the bounding box regression loss, we use the following:

(3) $$L_{box}\left(t_{i},t_{i}^{*}\right)=\sum_{j\in [0,4]}L_{1,smooth}\left(t_{i}\left(j\right)-t_{i}^{*}\left(j\right)\right),$$

where the robust loss function (smooth $L_{1}$) is given by the following.

(4) $$L_{1,smooth}\left(x\right)=\left\{\begin{array}{cc} 0.5x^{2} & if|x|<1\\ |x|-0.5 & otherwise. \end{array}\right.$$

The term $p_{i}^{*}$ means that the box regression loss is only activated for positive anchors ($p_{i}^{*}$ = 1). The two terms are normalized by $N_{cls}$ and $N_{box}$, which denote the number of images per batch (we have set $N_{cls}=16$) and the number of anchor locations. The hyper-parameter λ controls the balance between the two task losses. In our experiments, we used λ=0.5 as the precise bounding box is of less interest in our case. Finally, we used a base learning rate of 0.00025.

### 5.4. Data Augmentation

In view of enlarging our training set, we incorporate several data augmentations, which we divide in two categories: (1) geometrical and (2) appearance based. Regarding the first group, we include random flips using the horizontal and vertical axis with a probability of 0.5. Furthermore, we apply a random rotation ranging from $-45^{\circ }$ to $45^{\circ }$ with a probability of 0.5. Finally, we create crops of 25×25 pixels. The hotspots in our datasets are ranging from circular blobs with a bounding box of 50×50 pixels to elongated blobs with a bounding box of approximately 50×100 pixels. Recall that the annotations were generated using SLIC-based segmentation and that the precise bounding boxes are therefore slightly ambiguous. To deal with this, we guaranteed that the crops are either centred or include at least two sides of the bounding box. In other words, we apply a ‘five-crop’: four crops from four different corners + a center crop. Regarding the appearance-based augmentations, we have integrated random lighting, saturation, contrast and brightness. We increased saturation and the contrast with a random factor between 1.0, i.e., a preservation of original value and 1.5. We, thus, only permitted saturation and brightness increases and excluded decreases.

### 5.5. Post-Processing

As mentioned in the previous section, the bounding boxes for the hotspots are slightly ambiguous as hotspots can be smeared out or appear in a set of multiple hotspots located close to each other. For humans, it is challenging to define the precise bounding box; thus, it is also hard to define thresholds for the SLIC-based segmentation. As a result, the CNN model will also be unconfident about the precise location of the bounding box. Normally, several potential bounding boxes will be indicated by the CNN. Therefore, we apply a non-maximum suppression by only picking the bounding box with the highest confidence score in the set of overlapping bounding boxes. This approach does not jeopardize the effectiveness of hotspot detection as the precise bounding box is secondary to detection itself.

## 6. Evaluation and Discussion

The evaluation of the hotspot detection in thermal images has been conducted using the data discussed in Section 4. For each predicted bounding box, the overlap with the ground truth bounding box is measured using the intersection over union (IoU), which is computed as the area of overlap divided by the area of union. Then, we use an IoU threshold of 0.5 to determine whether the detection is a true positive (IoU higher than 0.5) or a false positive (IoU lower than 0.5). Given this IoU threshold, we subsequently calculate the true positive rate (TPR) or recall, which is defined as the ratio between the correctly classified hotspots (TP = true positives) and the total number of hotspots in the dataset (sum of the TP and FN = false negatives).

(5) $$\mathrm{TPR}=\frac{\mathrm{TP}}{\mathrm{TP}+\mathrm{FN}}.$$

To evaluate whether our model results in a lot of false positives, we generated a dataset containing 8924 *healthy* solar panels, which is a realistic amount for a large PV site. Subsequently, we compute the false positive rate (FPR), which is defined as the ratio between the number of *healthy* solar panels wrongly categorized as anomalous (FP = false positives) and the total number of solar panels that were tested (sum of FP and TN = true negatives).

(6) $$\mathrm{FPR}=\frac{\mathrm{FP}}{\mathrm{FP}+\mathrm{TN}}=\frac{\mathrm{FP}}{N}.$$

In total we have set up three different experiments. In the first experiment, we trained the network by using a subset of data from 1 PV site and evaluated it on a disjoint subset from the same PV site. In the second experiment, we trained the network using data acquired from a few PV sites and evaluated it on data from unseen PV sites. Finally, in the third experiment, we aggregated data from all PV sites and evaluated it on (a disjoint subset of) one particular PV site.

### 6.1. Experiment 1

For the first experiment, we use one dataset to train the R-CNN model and evaluated the performance of the model using the same dataset. We split the entire dataset in a training set and a test set as follows: 3/4 of the dataset is used for training and 1/4 is used for testing. We, thus, guarantee that no anomaly from the latter is present in the former. The same amount of negative samples were added to the training set as well.

The three largest PV sites were used for this experiment, as the other PV sites had too few positive samples to reliably train a neural network: *Hokota*, *Serramanna* and *Montalto di Castro*, which have, respectively, 1144, 316 and 166 hotspots present. The PV sites contain more potential hotspots, but we selected only those with a delta temperature of at least 5° to avoid using ambiguous anomalies in the evaluation. Note that we trained three different models using three different datasets.

In Table 2, the results of this first experiment are listed. They show that we achieve a TPR or recall of more than 90% for the three data sets. For the same threshold on the confidence score of the AI model, we obtain very few positives, especially for the datasets Hokota and Serramanna, which have a FPR of 3.4% and 0.4%, respectively. The higher FPR on the Montalto di Castro site is probably due to fewer positive samples in the training set compared to the other PV sites.

### 6.2. Experiment 2

To further verify the generalization of our CNN network, we have set up a second experiment for which we make sure that no PV site from the training dataset is present in the testing dataset. Thus, we use all anomalies from the PV sites *Serramanna*, *Hokota* and *Omuta* to train the R-CNN model and we evaluated it on two unseen PV sites *Montalto di Castro* and *Kumagaya*. An additional challenge was imposed by the fact that the solar panels from the evaluation datasets have a ‘vertical’ orientation with respect to the flight direction, as opposed to the ‘horizontal’ orientation from the training dataset.

In Table 3, the results of the second experiment are listed. For the site *Montalto di Castro*, the TPR slightly decreased from 90.2% to 82.3%, which is not surprising given the fact that no single anomaly from this PV site was present in the training set. The TPR on the *Kumagaya* dataset exceeds once again 90%, indicating that the aggregated model is generalizing pretty well for unseen data. The FPR is slightly higher than was the case in the first experiment (for the datasets *Hokota* and *Serramanna*), but that is also a logical consequence from the fact that the AI model was not trained specifically on these datasets. Note that we can lower the confidence threshold for our AI model to obtain fewer false positives, but that would also result in a lower TPR. Moreover, note that the same FPR is listed twice here as we considered the same model 4.

### 6.3. Experiment 3

Finally, for the third experiment we aggregated the data from all PV sites and divided it again in two disjoint sets: a training and a test set. The training set, therefore, contains images from all of the available PV sites. We evaluated the CNN model on a test set containing anomalies from the Serramanna dataset. Note that we still guarantee that no anomaly from this Serramanna test set is present in the training set. The results are listed in Table 4.

**Figure 9:**
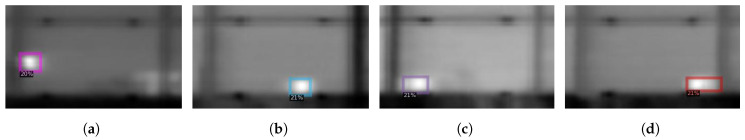
Four different hotspots (**a**–**d**) detected using our region-based CNN algorithm on the dataset acquired at Omuta, Japan.

**Figure 10:**
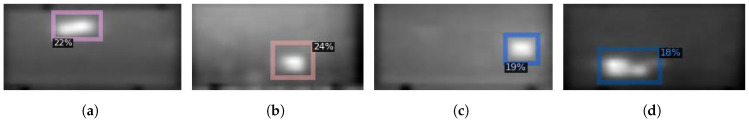
Four different hotspots (**a**–**d**) detected using our region-based CNN algorithm on the dataset acquired at Serramanna, Italy.

**Figure 11:**
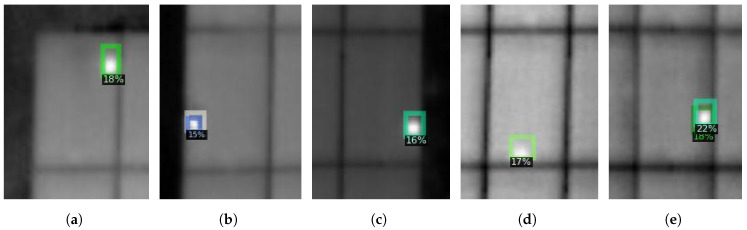
Five different hotspots (**a**–**e**) detected using our region-based CNN algorithm on the dataset acquired at Kumagaya, Japan.

**Figure 12:**
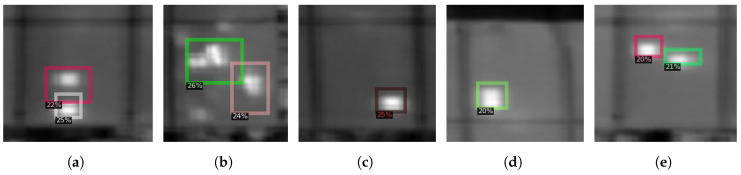
Five different hotspots (**a**–**e**) detected using our region-based CNN algorithm on the dataset acquired at Montalto Di Castro, Italy.

**Figure 13:**
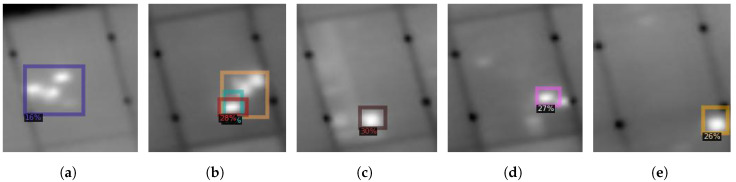
Five different hotspots (**a**–**e**) detected using our region-based CNN algorithm on the dataset acquired at Madrid, Spain.

As observed, TPR increased from 92.3% to 96.2%, corresponding to, respectively, 72 and 75 out of 78 anomalies from the test sets that are now detected, while the FPR of 2.4% on the negative set remains very low. This experiment demonstrates that we can increase the TPR when the R-CNN is trained using more date from more PV sites, while keeping the number of false positives very low, which is of course what was expected. The result of this last experiment is definitely the one that is most representative as it used data from all the different PV sites. One can expect the performance of this model to improve more when the model is retrained using even more data. Some visual results of this trained model on the different datasets are visualized in Figure 9, Figure 10, Figure 11, Figure 12 and Figure 13.

### 6.4. Comparison with the State-of-the-Art

In this section, we compare our results with three different state-of-the-art methods. The first method, proposed by Akiyama et al. [2], is based on direct current (DC) and is able to monitor PV sites on the solar panel level, making it suitable as a reference to compare our method with. The second method, presented by Pierdicca et al. [10], uses thermal imagery and is based on the VGG-16 network architecture, consisting of five convolutional blocks followed by a fully connected classifier. They apply the network on images of multiple solar panels that are resized to 224 × 224 pixels. In other words, the method does not segment or crop the solar panels prior to the detection of anomalies. Given this fundamental difference with our own approach, it makes it interesting to compare both.

Finally, Alves et al. [13] proposed a technique based on CNNs and thermographic images to detect and classify faults in solar panels. Their proposed network takes as input images with dimensions 40×24 and consists of four convolutional layers followed by three fully connected layers. Thus, this method uses images from solar panels that are pre-cropped from the background; therefore—to use it in an operational framework—the solar panels should first be detected. In this paper, we assumed that the solar panels were pre-detected using our method described in [15]. The approach closely resembles the one of our own method. The main difference lies in the design of the network architecture, yet making it another interesting comparison.

As before, we use the same metrics, i.e., true positive rate and false positive rate, to compare different methods. Some of the authors also use the accuracy (ACC) as additional metric (some of them refer to it as the conformity ratio), which is defined as follows.

(7) $$\mathrm{ACC}=\frac{\mathrm{TP}+\mathrm{TN}}{\mathrm{TP}+\mathrm{FP}+\mathrm{FN}+\mathrm{TN}}.$$

The accuracy is not the best metric for this use case as there will always be an uneven class distribution: There are many more ‘healthy’ solar panels than there are ‘faulty’ solar panels. Nonetheless, we computed the accuracy for our method as well. To compare with the aforementioned methods, we take the evaluation of our fifth model (cfr. Table 4), trained and tested using an aggregated dataset. The comparison with the three other state-of-the-art methods is presented in Table 5.

Table 5 reveals that the accuracy for our method is the highest among all methods while the false positive rate is the lowest. In addition, the dataset on which we tested our model is the most representative as we used a large PV site containing nearly 9000 solar panels.

The method of Akiyama et al. is also performing quite well as it does not miss any anomalies. The false positive rate is twice as high as our method, but nevertheless it remains rather low. We believe that the combination of the DC-based method and our thermographic-based method would be complementary and would result in much better results.

As expected, the method of Alves et al. produces similar results as our method. Still, the true positive rate for our method is 0.032 higher, whereas the false positive rate is more than three-times lower (0.024 versus 0.08). It must be mentioned that the method of Alves et al. also classifies the anomalies based on their thermographic signature, which is a clear advantage over our method.

The method of Pierdicca et al. is clearly the lowest performing one. The true positive rate of 0.672 is rather low and the false positive rate of 0.171 is very high. These poor results are not surprising as the algorithm does not determine a region of interest in advance but takes as input an image of 224 × 224 pixels containing multiple solar panels, which is clearly a more difficult problem. We must, therefore, be careful with the comparison as in our case and in the case of Alves’ method, we did not integrate the localization of solar panels in the evaluation while it should somehow be reflected in the there. However, in [15], we concluded that our solar panel detection achieved a true positive rate of 0.97, so the two methods combined are still performing much better than the method of Pierdicca et al.

## 7. Conclusions

In this paper, a novel anomaly detection system for large PV sites using drone-based imaging was presented. A clear overview was provided in the main steps including orthophoto generation, solar panel detection and anomaly detection. In addition, a thorough description and evaluation was presented on the topic of PV anomaly detection in thermal images. Experiments demonstrated that we were able to train high-performance models achieving a true positive rate of more than 95%. For ‘unseen’ PV sites, the true positive rate still exceeds 90% when the PV site is similar to one or more sites from the training set, whereas for ‘unseen’ PV sites having a dissimilar appearance, the true positive rate is still higher than 80%. Finally, the corresponding false positive rate for a model trained using all of the available PV sites and tested on almost 9000 solar panels was very low (between 2% and 3%). Compared to the best state-of-the-art methods, the experiments revealed that we achieve a slightly higher true positive rate for a substantially lower false positive rate, while it tested for a more realistic dataset containing almost 9000 solar panels.

## Figures and Tables

**Table 1:** The specifications of the different datasets used for training and validation.

Dataset	Type	Orientation	Hotspot	Multi-Hotspots
Omuta (Japan)	ground	horizontal	801	124
Kumagaya (Japan)	ground	vertical	56	71
Madrid (Spain)	rooftop	oblique	211	377
Serramanna (Italy)	rooftop	horizontal	5	4140
Hokota (Japan)	ground	horizontal	4793	2136
Montalto di Castro (Italy)	ground	vertical	197	304

**Table 2:** Experiment 1: True positive rate (TPR) and false positive rate (FPR) for hotspot detection in thermal images. The R-CNN model was trained using one dataset and evaluated on a disjoint subset of the same dataset.

Dataset	Model	P (Train)	P (Test)	N (Test)	TP	TPR	FP	FPR
Hokota	1	857	287	8924	275	**0.958**	307	**0.034**
Serramanna	2	238	78	8924	72	**0.923**	33	**0.004**
Montalto di Castro	3	125	41	8924	37	**0.902**	1484	**0.166**

**Table 3:** Experiment 2: True positive rate (TPR) and false positive rate (FPR) for hotspot detection in thermal images. The R-CNN model was trained using an aggregated dataset and evaluated on a test dataset with ‘unseen’ PV sites.

Dataset	Model	P (Train)	P (Test)	N (Test)	TP	TPR	FP	FPR
Montalto di Castro	4	2100	124	8924	102	**0.823**	1183	**0.133**
Kumagaya	4	2100	53	8924	49	**0.925**	1183	**0.133**

**Table 4:** Experiment 3: True positive rate (recall) for hotspot detection in thermal images. The R-CNN model was trained using an aggregated dataset from different PV sites and evaluated on a dataset from one particular PV site for which examples were also present in the training set.

Dataset	Model	P (Train)	P (Test)	N (Test)	TP	TPR	FP	FPR
Serramanna	5	2024	78	8924	75	**0.962**	213	**0.024**

**Table 5:** Qualitative comparison of our anomaly detection algorithm with state-of-the-art methods.

Method	P	N	Total	TP	FN	FP	TN	TPR	FPR	ACC
Akiyama [2]	52	830	882	52	0	45	785	1.0	0.054	0.949
Alves [13]	1275	1275	2550	1186	89	102	1173	0.93	0.08	0.925
Pierdicca [10]	652	662	1314	438	214	113	549	0.672	0.171	0.751
Ours	78	8924	9002	75	3	213	8711	0.962	0.024	0.968
